# Adsorptive Elimination of a Cationic Dye and a Hg (II)-Containing Antiseptic from Simulated Wastewater Using a Metal Organic Framework

**DOI:** 10.3390/molecules29040886

**Published:** 2024-02-17

**Authors:** Nilanjan Roy, Chanchal Das, Mohuya Paul, Jungkyun Im, Goutam Biswas

**Affiliations:** 1 Department of Chemistry, Cooch Behar Panchanan Barma University, Cooch Behar 736101, West Bengal, India; roynilanjancob@gmail.com (N.R.); chanchaldas453@gmail.com (C.D.); 2 Department of Electronic Materials, Devices and Equipment Engineering, Soonchunhyang University, Asan 31538, Republic of Korea; mohuyapaul678@gmail.com; 3 Department of Chemical Engineering, Soonchunhyang University, Asan 31538, Republic of Korea

**Keywords:** metal organic framework (MOF), wastewater management, natural ions, safranin O and merbromin, dyes and pharmaceutical wastes, adsorption

## Abstract

Several types of pollutants have acute adverse effects on living bodies, and the effective removal of these pollutants remains a challenge. Safranin O (a biological dye) and merbromin (a topical mercury-containing antiseptic) are considered organic pollutants, and there are only a few reports on their removal. Synthesized and well-characterized (through PXRD, FTIR, FESEM, and EDS analysis) MOF-5 was used for the first time in the removal of safranin O and merbromin from simulated wastewater and real wastewater. In both cases, MOF-5 effectively removed contaminants. We found that in simulated wastewater, the highest efficiency of removal of safranin O was 53.27% (for 15 mg/L) at pH 10, and for merbromin, it was 41.49% (for 25 mg/L) at pH 6. In the case of real wastewater containing natural ions (Na^+^, K^+^, F^−^, Cl^−^, SO_4_^2−^, PO_4_^3−^, Mg^2+^, and Ca^2+^) and other molecules, the removal efficiencies of these two dyes decreased (34.00% and 26.28% for safranin O and merbromin, respectively) because of the presence of other ions and molecules. A plausible mechanism for the removal of these pollutants using MOF-5 was proposed.

## 1. Introduction

**Figure 1:**
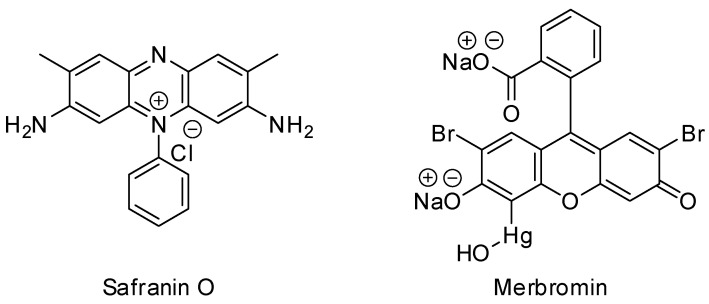
Structures of safranin O and merbromin.

The amount of pure drinkable water decreases daily, for both natural and artificial reasons. Industrial development is a major contributor to water pollution. Since the very beginning of the industrial revolution, as the industrial sectors have expanded every year, the amount of various hazardous chemicals contaminating groundwater has increased rapidly [1,2]. Different types of dyes are pollutants that add up to a serious issue of water pollution [3]. There are various types of dyes that are used in different industries, especially in the textile industry, broadly divided according to the (a) method of application and (b) chemical structure [4]. On the other hand, based on charge, dyes can be classified into three classes: (a) cationic dyes (e.g., rhodamine B, methylene blue, and rhodamine 6G), (b) anionic dyes (e.g., Congo red, methyl orange, and acid fuchsin), and (c) neutral dyes (e.g., Giemsa stain) [5,6]. In general, anionic dyes tend to be acidic, whereas cationic dyes are basic in nature [7]. Anionic dyes are utilized for the modification of acrylic, polyamide, and polypropylene fibers, whereas cationic dyes are frequently used in the dyeing of acrylic, wool, and silk fibers [5]. There are also different pharmaceutical pollutants, such as staining dyes, antibiotics, antimicrobial medicines, and other harmful drugs, which are also responsible for serious water pollution and severe negative impacts on the environment. Safranin-O and merbromin (Figure 1) are hazardous pollutants [8]. Safranin O (or safranin) is a well-known cationic dye that is frequently used to stain cells in laboratories and hospitals [9]. However, safranin O also has detrimental effects on the cardiovascular system and other serious health problems, including methemoglobinemia, cyanosis, spasms, and severe eye and skin irritation [10].

On the other hand, merbromin, a mercury-ion containing pharmaceutical waste (which is a sodium salt of 2,7-dibromo-4-hydroxymercurifluorescein), is typically used as an antiseptic, mostly for external treatments of cuts and scrapes [11]. Although many of us are unaware of its hazardous effects, we apply it to different injuries, minor wounds, and burns [11]. It is responsible for gastrointestinal problems, such as nausea and vomiting like some other drugs and pharmaceuticals [12]. Merbromin can result in high mercury concentrations in the blood, urine, and other organs, as well as severe digestive system damage, renal failure, and toxicity to other organs [11].

Therefore, it is necessary to eliminate safranin O and merbromin from water to prevent their adverse effects on many living systems. Safranin O and merbromin were chosen as the main hazardous pollutants for this investigation. Few methods have been reported for removing these two compounds from water.

Currently, there are different methods for the removal of these contaminants, such as osmosis [13], photocatalytic degradation [14], membrane filtration [15], electrochemical process [16], electrolysis [17], organic processes [18], and adsorption [19,20]. However, techniques other than adsorption are expensive, time-consuming, and complex, with poor recycling efficiency. Adsorption is considered an effective, convenient, and reliable technology because of its simple and straightforward method and operation and high proficiency, even at extremely low concentrations. Several materials have been reported to remove these toxic pollutants, including nanomaterials [21,22], minerals [23], quantum dots [24], metal organic frameworks (MOFs) [25], and covalent organic frameworks (COFs) [26]. Various MOFs employed for wastewater treatment have been reported in the literature. MOFs have a variety of intriguing distinctive traits, including a large surface area, numerous adjustable pores, and tunable surface properties [27]. These features make MOFs promising tools for adsorption, separation, drug delivery, ion exchange, storage, and catalysis [28,29,30]. Various adsorbents have been reported to remove organic/organometallic molecules. Among these, some materials suffer from several drawbacks, such as complicated or costly synthesis. Like zeolites, which require an inorganic or organic template for their preparation, solvents are the major templating molecules for the synthesis of MOFs [31]. In addition, many materials, such as different aerogels and nanomaterials, have been reported to be less efficient in wastewater treatment compared with MOFs [32,33,34,35,36,37].

MOF-5 (chemical formula: Zn_4_O_13_(C_8_H_4_)_3_), also known as IRMOF-1, a well-known MOF, has vast applications because of its high porosity, unique structure, and thermal stability. It has a three-dimensional structure consisting of Zn_4_O clusters and terephthalate ligands [38]. The size and porosity (or pore volume) of an adsorbent determine its adsorption efficiency.

In the present context, we prepared MOF-5 following a procedure reported in the literature with slight modifications and characterized it by FT-IR, powder XRD (PXRD), FESEM, and EDS analysis. The as-prepared MOF-5 was used to remove safranin O and merbromin from simulated and real wastewater via adsorption. This is the first time MOF-5 has been used to eliminate these two pollutants.

## 2. Results and Discussion

### 2.1. Characterizations of the Synthesized MOF-5

#### 2.1.1. PXRD Analysis

**Figure 2:**
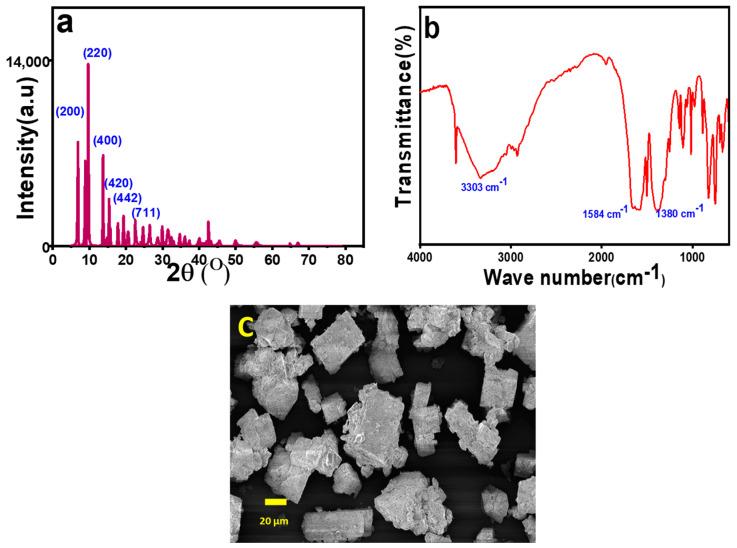
(**a**) PXRD pattern, (**b**) FTIR spectra, and (**c**) FESEM image of MOF-5.

Figure 2a shows the PXRD pattern of the synthesized MOF-5. All characteristic peaks appear at 2θ values of 6.86°, 9.71°, 13.6°, 15.3°, 22.5°, 24.9° and 26.3°, which represent the corresponding crystal planes of (200), (220), (400), (420), (442), (711), and (731), respectively. These are very similar to the reported MOF-5 PXRD diffraction data in the literature [39,40] and closely match the file number of the Joint Committee on Powder Diffraction Standards (JCPDS) 36–1451 [41]. From these data, it can be concluded that MOF-5 has a cubic unit cell crystal structure. The sharp and large intense peaks represent the high crystallinity of the adsorbent, which is consistent with the FESEM image (Figure 2c).

#### 2.1.2. FT-IR Analysis

The skeletal arrangement of MOF-5 was validated using FT-IR spectroscopy. The stretching vibrations of the -COO group in the terephthalic acid linker had symmetric and asymmetric stretching peaks at 1380 cm^−1^ and 1584 cm^−1^ in the FT-IR spectra (Figure 2b). This stretching vibration was different from the infrared spectrum of the terephthalic acid ligand, which appeared at 1660 cm^−1^. The peaks corresponding to >C=O exhibited a noticeable red shift. This is due to the formation of terephthalate and Zn^2+^ coordination of the ligand -COO, that is, COO-Zn^2+^. This makes the carbonyl electron distribution relatively uniform and lowers the electron cloud’s density [42]. The broad line at 3000–3500 cm^−1^ indicates the presence of water molecules coordinated to the metal center [43]. The sharp peak at 1502 cm^−1^ represents the C=C vibration that is present in the linker [40]. Several small peaks within the ranges of 1250–950 cm^−1^ and 800–650 cm^−1^ represent the in-plane and out-of-plane vibrations of the aromatic C-H bonds [44]. When compared with the data mentioned in the literature, it can be justified that pristine MOF-5 crystals were formed in the present synthesis.

#### 2.1.3. FE-SEM Analysis

The morphology of MOF-5 was investigated by FESEM. In the present work, it was found that the average size of the prepared MOF-5 ranged from 10 to 20 μm (Figure 2c) of the prepared MOF-5. As shown in Figure 2c, all MOF-5 particles were cubic in shape. Mirsoleimani-Azizi et al. synthesized cubic MOF-5 with an average diameter of approximately 550 nm using zinc acetate at room temperature [45]. Cubic MOF-5 with a crystal size of 5–25 μm was synthesized by Son et al. using a rapid sonochemical method [46]. Uniform cubic MOF-5 crystals, 20–25 μm in size, were also prepared through a microwave heating solvothermal route using 1-methyl-2-pyrrolidone as a solvent by SikChoi et al. [47]. Zhao et al. synthesized cubic MOF-5 monocrystals with diameters of 40–60 µm [48].

#### 2.1.4. EDS Analysis

The elemental composition of MOF-5 was analyzed using EDS. The elemental mapping of the uniform distribution of C, O, and Zn in Figure S1 demonstrates how the skeleton function of MOF materials can prevent the aggregation of metal particles and ensure that the appropriate metal elements are equally dispersed, which is very similar to the data reported in the literature [49].

### 2.2. Effect of pH

**Figure 3:**
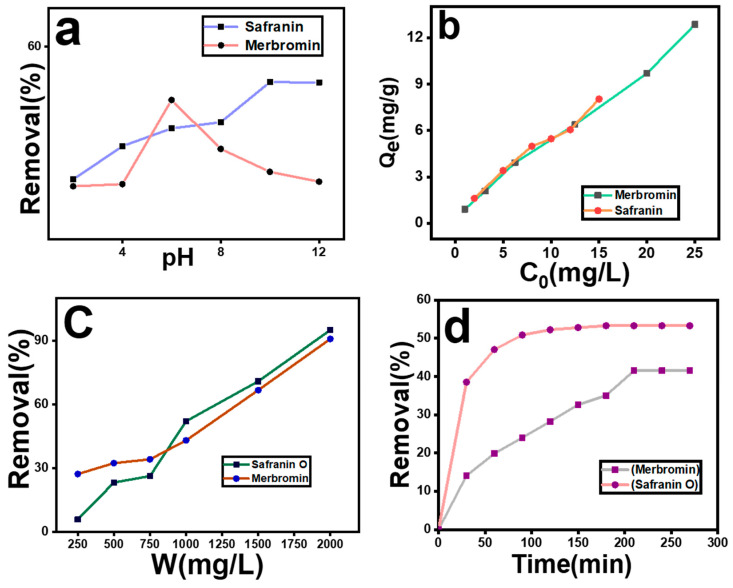
Effect of (**a**) pH, (**b**) initial concentration of contaminant, (**c**) amount of adsorbent, and (**d**) effect of contact time on removal percentage.

Figure 3a shows the variation in percentage removal under different pH conditions for both safranin O and merbromin. The maximum removal efficiency was observed at pH 10 (53.27%) for safranin O and at pH 6 (41.49%) for merbromin. The point of zero charge (PZC) for the MOF-5 was at pH 4.6, which matches well with the reported value [50]. Hence, the surface of MOF-5 becomes negatively charged above pH 4.6 and positively charged below this pH [50]. Safranin O is a cationic azo dye, while merbromin (disodium organomercuric salt) contains a negatively charged exterior owing to the presence of –O^−^ and –COO^−^ groups. This information is consistent with that observed in the current study on the effect of pH on pollutant removal. Hence, for safranin O, removal increased as the surface became increasingly negative. For merbromin, an increase was observed as the pH increased from 2 to 6, and above pH 6, the removal efficiency decreased because of the increasing repulsive interaction between the negatively charged merbromin and MOF-5. Significant electrostatic interactions between the adsorbent and adsorbate occurred during adsorption. In addition, there may be a weak noncovalent force of attraction between merbromin and MOF-5, for which it was adsorbed. There may be substantial H bonding between the OH and –COOH groups of merbromin (at pH 6) and the adsorbed –OH on MOF-5 at low concentrations of –OH groups on MOF-5 (at pH-6).

### 2.3. Effect of Adsorbent Dose

The mass of the adsorbent is also a determining factor, because it provides adsorption sites for adsorbate molecules. Figure 3c shows the variation in the removal percentage with the adsorbent dosage (W). The removal efficiency of the pollutants increased with an increase in the amount of adsorbent (from 250 to 2000 mg/L) and was found to be maximum (95.01% and 90.80% for safranin O and merbromin, respectively) at an adsorbent concentration of 2000 mg/L. This result was obvious, as the increase in the amount of adsorbent provided a greater number of adsorption sites for capturing the adsorbate molecules during the adsorption process, which ultimately enhanced the adsorption [49].

### 2.4. Effect of Contact Time and Adsorption Kinetics

As the adsorption time increased, the pollutant was increasingly adsorbed on MOF-5. The equilibrium times for safranin O and merbromin were 180 min and 210 min, respectively. After equilibrium was reached, the adsorption percentage remained almost unchanged. When equilibrium was reached, the percentage removal was 53.27% and 41.49% for safranin O and merbromin, respectively (Figure 3d). This result was obvious, because when equilibrium was reached, all the adsorption sites were closed, and there were no vacant adsorption sites.

**Figure 4:**
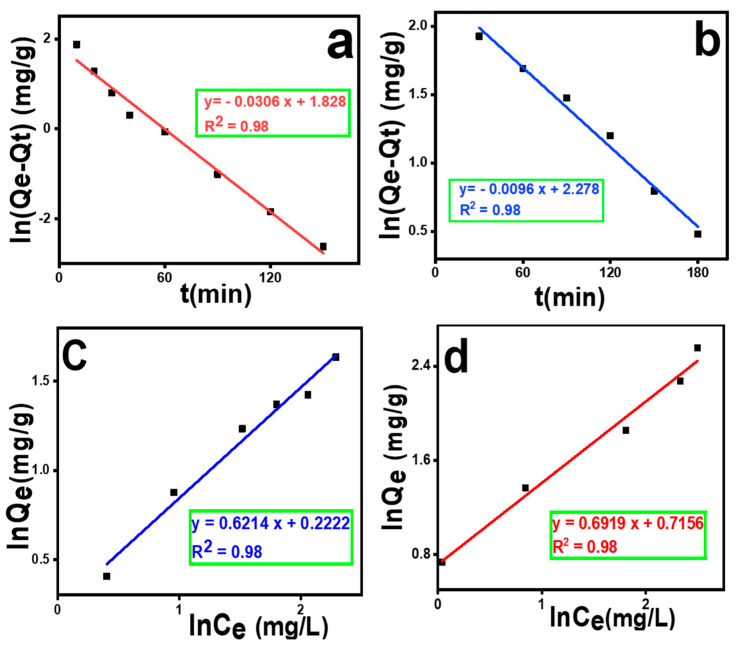
(**a**) Pseudo-first-order plot of safranin O removal, (**b**) pseudo-first-order plot of merbromin removal, (**c**) Freundlich isotherm plot of safranin O adsorption, and (**d**) Freundlich isotherm plot of merbromin adsorption.

For the kinetic study, the pollutants were adsorbed on MOF-5 for 180 min for safranin O and 210 min for merbromin. The removal percentages in both cases were checked periodically, and the data are plotted in Figure 4a,b. The kinetic data fit well to the linear form of the pseudo-first-order model. All calculations and related figures are presented in Supplementary Materials (Table S1 and Figure S2). Here, for safranin O, as the value of R^2^ (=0.98) was close to unity and the value of the experimental adsorption capacity (Q_e_(ex) = 7.99 mg/g) was well matched to the calculated value (Q_e_(cal) = 6.22 mg/g), it can be concluded that in this case, a pseudo-first order should be the kinetic model. Meanwhile, in the case of merbromin, R^2^ was 0.98, and here, Q_e_(ex) = 10.37 mg/g, which was matched well with the theoretical one (Q_e_(cal) = 9.75 mg/g) for the pseudo-first order. Here, R^2^ is the correlation coefficient, as reported in the literature [51]. According to the literature, our experimental data fit well with the pseudo-first-order kinetic model, as evidenced by the highest R^2^ value [51,52].

### 2.5. Effect of Adsorbate Dosage and Isotherm Modeling

The variation in the initial concentration (C_o_) of waste contaminants is another important factor in the sorption process. The equilibrium adsorption capacity (Q_e_) was found to be maximum (8.19 mg/g) at the 15 mg/L concentration and minimum (1.61 mg/g) for the 2 mg/L solution of safranin O. Meanwhile, in the case of merbromin, the equilibrium adsorption capacity (Q_e_) was found to be maximum (12.22 mg/g) at a higher concentration (25 mg/L) and minimum (0.09 mg/g) at a lower concentration (1 mg/L) (Figure 3b). This occurs because at higher concentrations of pollutant molecules, there is a higher probability of collisions with adsorption sites on the adsorbent surface. Again, the mass transfer improves during adsorption, which can decrease the mass transfer resistance and hence increase the adsorption capacity [53,54].

Isotherm modeling is important for this analysis, as it provides information regarding the isotherm adsorption behavior for the removal process of these contaminants. Five isotherm models (Langmuir isotherm, Freundlich isotherm, Temkin isotherm, Elovich isotherm, and Dubinin–Radushkevich isotherm models) were investigated to determine the adsorption process. The results of the experiment are shown in Figure 4c,d, Figures S3 and S4 and Table S2. From the data, it can be concluded that the best-fitting model for both cases was the Freundlich isotherm. Thus, the adsorption was multilayered, with K_F_ = 1.24 [mg. g^−1^ (mg L^−1^)^−0.6214^] (for safranin O) and 2.04 [mg. g^−1^ (mg L^−1^)^−0.6919^] (for merbromin), and n values were 1.60 (for safranin O) and 1.44 (for merbromin). Linear fitting was performed using the R^2^.

Different Q_max_ values for dye removal by MOF-5 are reported in Table 1 (if we assume that the removal follows the Langmuir isotherm model, then only the Q_max_ values were 8.19 mg/g and 22.22 mg/g for safranin O and merbromin, respectively (Table S2)). From the table, it can be concluded that our prepared MOF-5 also works as well as the other reported MOF-5s, and that it is even superior to some other MOF-5s.

Overall, it can be concluded that MOF-5 had certain advantages over other different adsorbents. It has also been found that MOFs are more or similarly efficient in comparison to other kinds of adsorbents (e.g., nanomaterials, bio adsorbents, graphene oxide, carbon nanotubes, and aerogels) that are used for wastewater treatment. Comparisons between the Q_max_ values of different adsorbents for the removal of different dyes are reported in Table 2 (to the best of our knowledge, merbromin removal has not yet been reported).

### 2.6. Thermodynamics of Adsorption

**Figure 5:**
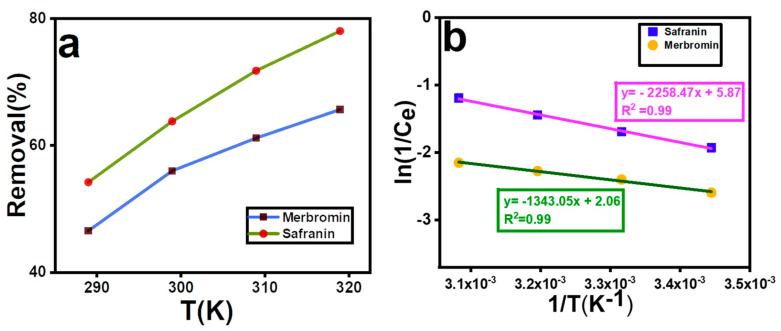
(**a**) Effect of temperature on removal percentage, (**b**) plot of ln(1/C_e_) vs. (1/T).

The adsorption removal efficiency with respect to temperature is shown in Figure 5a, and the data are represented in Table S3. The removal efficiency increased with increasing temperature in both cases. The data were plotted as [ln (1/C_e_) vs. temperature (1/T)] (Figure 5b), and the thermodynamics of adsorption were modeled using linear regression curves. The R^2^ value was higher than 0.98, which proved that the curves were well fitted. ΔH values were calculated to be 18.77 kJ/mol and 11.16 kJ/mol for safranin O and merbromin, respectively, from Figure 5b (overall results are displayed in Table S3). A positive ΔH value indicates that both adsorptions are endothermic in nature, and a negative ΔG value implies that the adsorption is spontaneous [59]. The ΔG value is important because it indicates whether the adsorption is physisorption or chemisorption. If the ΔG value is negative and ranging from 0 to 20 kJ/mol, then it is physisorption, and if it is negative within the range of 80 kJ/mol to 400 kJ/mol, then it is chemisorption [60]; however, the ΔG° value varied from −3.8 to −4.2 kJ/mol for safranin O and from −3.4 to −3.8 kJ/mol for merbromin, and in both cases, the negative ΔG value was lower than 20 kJ/mol; hence, the adsorption process was physisorption [49,61]. Here, Δ*S* was highly positive, indicating that during the adsorption process, the degrees of freedom increased at the solid–liquid interface [45].

### 2.7. Effect of Natural Ions and Molecules Present in Real Water

**Figure 6:**
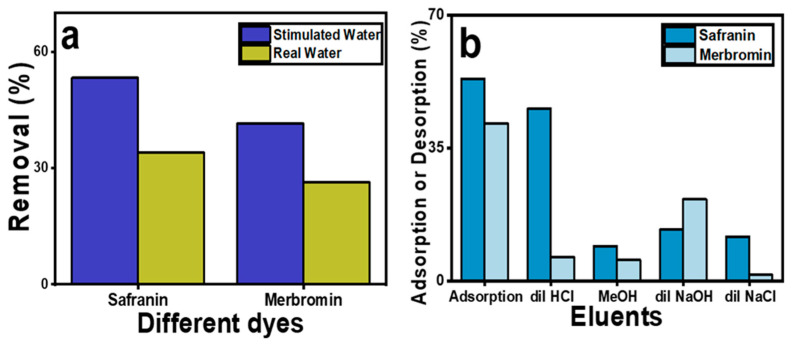
Comparison of removal percentage in (**a**) simulated wastewater and surface water containing various ions; (**b**) desorption of the dyes in different eluents.

To determine the impact of other metal ions, anions, small molecules, and other microorganisms on the adsorption behavior of the two organic pollutants, an adsorption study in lake water was performed. The removal of various ions (Na^+^, K^+^, F^−^, Cl^−^, SO_4_^2−^, PO_4_^3−^, Mg^2+^, and Ca^2+^) from contaminated real water was performed using the same concentrations of the two pollutants. At room temperature and neutral pH, the adsorption percentages of safranin O and merbromin were 34.00% and 26.28%, respectively. These values are lower than those in the simulated wastewater. These changes were due to several ions and other molecules being present in the real wastewater sample, which competed with these pollutants and reduced their adsorption capacity. Table S4 presents their amounts, and a comparison of the percentages of adsorption of the pollutants in the simulated wastewater and lake water is presented in Figure 6a.

### 2.8. Regeneration of Adsorbent

Figure 6b shows the desorption percentages of both dyes in the different solvents after a single desorption cycle. It was found that after 180 min, the maximum desorption percentage for safranin O was 45.4% in HCl, for merbromin, it was 21.6% in NaOH, and it then remained constant after further exposure. The desorption percentages of safranin O were found to be 13.6% for NaOH, 11.7% for NaCl, and 9.2% for methanol. This is acceptable, as safranin O is a cationic dye, and H_3_O^+^ can effectively replace safranin O, because it can form a stronger interaction with MOF-5 than with safranin O. Furthermore, NaOH and NaCl both form the same cation Na^+^, so their desorption ability is comparable because of their poor charge density due to their larger size compared to H_3_O^+^.

However, for merbromin, the desorption percentages were 6.3% in HCl, 1.7% in NaCl, and 5.6% in methanol. In the presence of NaOH, owing to the high pH of the eluent, the surface of MOF-5 became highly negatively charged (PZC for MOF-5 was 4.6), along with the formation of more OH^−^ ions within the solution. Therefore, OH^−^ ions can effectively replace the anionic merbromin from the MOF-5 surface compared to other conditions. As the desorption percentage was lower than 50%, further cycles were not performed.

### 2.9. Plausible Mechanism of Adsorption

MOFs can have both positive and negative charges on their surface depending on the pH, while safranin O is a positively charged dye, and merbromin is anionic in nature [62]. If MOFs have a positive charge, they can attract negative or anionic dyes, and if MOFs are negatively charged, they can attract positively charged dyes electrostatically. Therefore, electrostatic attraction is the predominant factor in the adsorption of MOFs [63]. Dyes can also be adsorbed via π-π interaction [64], hydrogen bonding [65,66,67], ion exchange [68,69], Lewis acid–base interactions [70,71,72], etc.

For electrostatic interactions, the MOF surface and the adsorbate should have opposite charges, so that they can easily be attracted to each other and hence adsorbed through a pure Coulombic attraction force. In the case of π–π-type interactions, noncovalent interactions occur between the two aromatic rings. Hazrati and Safari [73] reported that the sorption of reactive black 5 on a Cd-based MOF (TMU-8) is due to π–π interaction between the aromatic ring of reactive black 5 and the framework of the MOF. Again, an exchange of ligands or ions between the two intricate structures is found for the sorption of dyes onto MOFs via the ion exchange process. According to Yao et al. [68], adsorption occurs via ion exchange between the [(CH_3_)_2_NH_2_]^+^ ions that are present in JLU-Liu 39 and cationic dyes (methyl violet, methylene blue, and rhodamine B) [68]. According to Zhao et al., the presence of Ni^2+^ as a Lewis acid enhances the removal of Congo red onto GO/MOFs through a Lewis acid–base interaction [72]. There is also a mechanism called adsorption through hydrogen bonding, which has rarely been reported.

**Figure 7:**
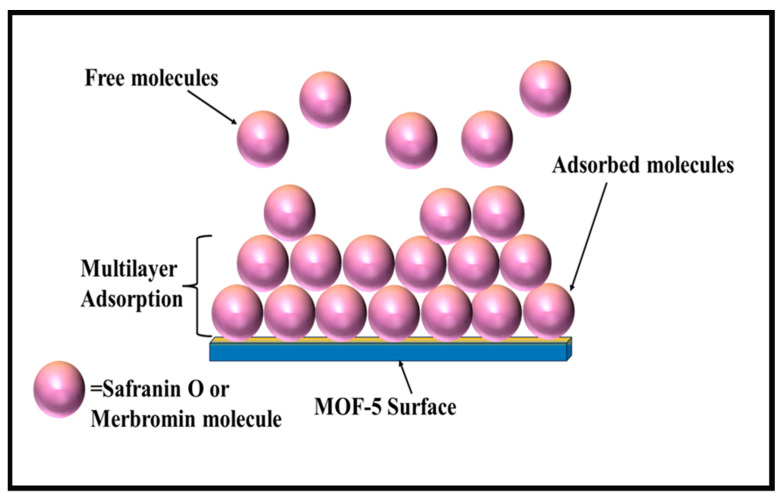
Plausible mechanistic pathway for multilayer adsorption of safranin O and merbromin on MOF-5.

According to the adsorption kinetics in the present context, MOF-5 is more useful and has a better adsorption capacity for cationic dyes (safranin O) than for merbromin. This may be explained by the electrostatic interactions between the negatively charged MOF-5 surface and the cationic dye (Figure 7). The carboxylate moiety of MOF-5 forms a negatively charged framework. At pH 10, the framework becomes negatively charged (PZC for MOF-5 was 4.6), thus enhancing the adsorption of safranin O.

In contrast, merbromin is electronically anionic, and at a lower pH, it becomes protonated and remains mostly neutral. Thus, at a lower pH, there might be a weak π-π-type interaction, but as the pH of the medium increases, there will be electrostatic repulsion between the merbromin molecule and the MOF-5 surface. Furthermore, there may be a major chance in the interaction between the aromatic ring of the ligand that is present in the MOF and the lone pairs of oxygen that are present in the merbromin dye at a lower pH. This π-π-type interaction was reported by Elsherbiny et al. [49] and was the main cause for the adsorption of merbromin onto MOF-5. As the π-π-type interaction is weaker than the pure electrostatic interaction, the removal percentage of merbromin is comparatively lower than that of safranin O.

## 3. Materials and Methods

### 3.1. Chemicals and Reagents

Safranin was purchased from Sigma-Aldrich, India, merbromin from Loba Chemie, Mumbai, India, and other common reagents were purchased from a local chemical company. All chemicals used in this study were of analytical grade and used without further purification. All solutions used in the adsorption experiments were prepared using Milli-Q water. The adsorption of these pollutants onto MOF-5 was also evaluated in a real wastewater sample, collected from Kochbihar Lake (Sagar Dighi), Cooch Behar, West Bengal, India, on 12 June 2023.

### 3.2. Preparation of MOF-5

MOF-5 was synthesized following a previously reported procedure [74] with some modifications. First, terephthalic acid (0.033 g, 0.2 mmol) was dissolved in 20 mL of DMF in a conical flask, and then, the zinc nitrate tetrahydrate (0.156 g, 0.6 mmol) solution (in 20 mL of DMF) was added with continuous stirring. The reaction mixture was heated in an oil bath at 110 °C for 24 h, cooled to room temperature, repeatedly washed with DMF followed by anhydrous chloroform, soaked for 24 h in chloroform, filtered, and vacuum-dried for 24 h. MOF-5 was then kept under vacuum for 2 h, while being activated at 105 °C.

### 3.3. Characterization Techniques

The common instruments used to perform the experiments were a Remi R-8C centrifuge, Remi orbital shaker (Model RS-36BL, Remi, Mumbai, India), Fisher Scientific Accumet pH meter (Model AB 250, Fisher Scientific, Loughborough LE11 5RG, UK), and Milli-Q Plant from Labconco Water Pro/Ro, Labconco, Kansas City, MO, USA. Skeletal analysis of MOF-5 was performed using Fourier transform infrared (FTIR) spectroscopy on a Benchtop Labtronics LT-4100, Labtronics, Welwyn Garden City AL7 1TW, UK, and crystallinity was investigated by powder X-ray diffraction (PXRD) on a Thermo Scientific ARL Equinox 1000, ThermoFisher Scientific, Waltham, MA, USA. Morphology, size, and elemental analyses were performed using field-emission scanning electron microscopy (FESEM) and energy-dispersive spectroscopy (EDS) (Zeiss EVO 18 from IIT Palakkad, Kerala, India). The experimental analysis was performed using a UV-vis spectrophotometer (Thermo Scientific Evolution 201, Fisher Scientific, Loughborough LE11 5RG, UK).

### 3.4. Adsorption Isotherm Model

Adsorption isotherm modeling is an important parameter. Numerous adsorption isotherm models have been developed to fit the experimental adsorption equilibrium data. This study utilized commonly used solid–liquid adsorption isotherm models. The different adsorption isotherm models with their equations and other statistical parameters are plotted in Table S2 [61,75,76,77].

### 3.5. Adsorption Thermodynamics

For the thermodynamic study of adsorption, different authors have reported different techniques for calculating adsorption thermodynamic parameters [78,79,80,81]. In this regard, as adsorption follows the Freundlich model, we consider the equations reported in the literature [76,82].

(1) $$\ln\frac{1}{C_{e}}=\ln K_{o}-\frac{∆H}{RT}$$

ΔG^o^ = −nRT (2)

(3) $${\Delta S}^{o}=\frac{{\Delta H}^{o}-{\Delta G}^{o}}{T}$$

where C_e_ is the equilibrium adsorbate concentration (mg/L) in the solution, n is the fitting constant of the Freundlich exponent, K_F_ is the Freundlich empirical constant. R is the universal gas constant (8.314 J/(mol K)) and T is the temperature (K). From the slope of the plot of ln 1/C_e_ versus 1/T, the enthalpy change in adsorption (ΔH°) was calculated.

## 4. Experimental

### 4.1. Batch Adsorption Experiments

A comparative study of the adsorption behavior of MOF-5 towards safranin O and merbromin was performed using the batch method. To study the effect of the initial adsorbate concentration, different concentrations of safranin O (2–15 mg/L) and merbromin (1–25 mg/L) were prepared from 100 mg/L stock solutions of the two pollutants. The removal percentages of pollutants were also studied using different amounts of adsorbent, ranging from 250 to 2000 mg/L. Adsorption was studied in the pH range of 2–12 to determine the effect of pH on removal. The pH of the solution was adjusted using 0.1 M NaOH and 0.1 M HCl. Adsorption experiments were conducted at different temperatures (289, 299, 309, and 319 K) to determine the thermodynamic parameters of the process, such as the Gibbs free energy change (ΔG), enthalpy change (ΔH), and entropy change (ΔS).

Except where otherwise stated, the sorption experiment was carried out at room temperature (r.t.) using 15 mg/L of safranin O at pH 10 and 25 mg/L of merbromin at pH 6; the volume of the solution was 10 mL. In all the cases, the amount of MOF-5 was 10 mg, which remained the same unless otherwise stated. Safranin O and merbromin were mixed with MOF-5 separately for 180 min and 210 min, respectively, and shaken at 250 rpm in an orbital shaker from Remi (model number: RS-36BL, Remi, Mumbai, India). Aliquots were periodically collected, centrifuged, and monitored using a UV-Vis spectrophotometer (519 nm for safranin O and 505 nm for merbromin) to evaluate the progress of the reaction. Before the UV-Vis spectral scanning for the determination of the pollutant concentrations, centrifugation was performed every time on a Remi centrifugation (Remi R-8C, Remi, Mumbai, India) at 1200 rpm for 10 min to extract MOF-5 from the solution.

The adsorption percentage and capacity (q_t_) were calculated using Equations (4) and (5), respectively.

(4) $$Adsorption\;(\%)=\frac{\left(C_{0}-C_{t}\right)}{C_{0}}\times 100$$

(5) $$q_{t}=\frac{\left(C_{0}-C_{t}\right)}{m}\times V$$

where C_o_ and C_t_ are the dye concentrations (mg/L) initially and after adsorption, respectively, and V and m are the volume (L) of the solution and mass (g) of MOF-5, respectively.

Because the adsorption process was performed in an aqueous medium, the aqueous stability of MOF-5 was an important issue. In many studies, it was mentioned that MOF-5 showed considerable stability in aqueous media even after 24 h, and as the equilibrium of adsorption was reached within 24 h in the present study, in the presence of water, the stability and the adsorption procedure were not hampered. For example, Elsherbiny et al. [49] concluded from the TGA data that Zn-BDC (MOF-5) is considerably stable upto 425 °C, which could be advantageous for the removal of pollutants at high temperatures. They also performed a test of stability in water, and the results were satisfactory. Therefore, MOF-5 could be used to treat wastewater. There are other examples too, such as Mohammadi et al. [56] used MOF-5 to remove malachite green from simulated wastewater.

### 4.2. Regeneration of Adsorbent

In addition to adsorption, regeneration (or desorption) is an important mechanism by which MOF can be recovered and reused. This was studied after the adsorption process for both safranin O and merbromin was completed. In this regard, MOF-5 was initially well saturated with the adsorbate solutions (15 mg/L at pH 10 for safranin O and 25 mg/L at pH 6 for merbromin; volume in each case = 10 mL); thereafter, centrifugation and decantation were performed, followed by washing with Milli-Q water. For the regeneration step, the adsorbate-loaded MOF-5 was then mixed with 0.01 M HCl, 0.01 M NaOH, 0.01 M NaCl, and 99.8% MeOH separately and shaken till equilibrium [83].

The percentage of desorption was determined by the following equation (Equation (6)):

(6) $$Desorption\;\left(\%\right)=\frac{amount\;of\;dye\;desorbed}{amount\;of\;dye\;adsorbed}\times 100$$

## 5. Conclusions

In general, MOF-5 was effectively synthesized and characterized by FT-IR, PXRD, FESEM, and EDS using the reported data. The MOF-5 particles were cubic. Two organic pollutants, safranin O and merbromin, were removed successfully using MOF-5 from the simulated wastewater. The pollutant removal followed pseudo-first-order kinetics and the Freundlich adsorption isotherm model. Moreover, thermodynamic parameters demonstrated that adsorption occurs naturally as physisorption and is thermodynamically favorable (ΔG = − ve for both safranin O and merbromin). A reduction in the percentage of adsorption occurred when real wastewater containing various ions (Na^+^, K^+^, F^−^, Cl^−^, SO_4_^2−^, PO_4_^3−^, Mg^2+^, and Ca^2+^) was used. Safranin O and merbromin are widely used in biological staining and pharmaceuticals despite their hazardous effects. We hope that this removal study will be helpful in controlling water pollution that is caused by industrial and pharmaceutical issues as much as possible by adding this new method for the improvement of the environment.

## Figures and Tables

**Table 1:** Comparison of Q_max_ for different dye removals by MOF-5.

Adsorbate	Type of Dye	Q_max_ (mg/g)	References
Aniline blue	Cationic	55.34	[49]
Methylene blue	Cationic	51.81	[55]
Malachite green	Cationic	50.69	[56]
Methylene blue	Cationic	2.632	[49]
Safranin O	Cationic	8.19	This study
Congo Red	Anionic	769.23	[57]
Orange II	Anionic	10.01	[49]
Merbromin	Anionic	22.22	This study

**Table 2:** Comparison of Q_max_ for different dyes with MOFs and other adsorbents.

Adsorbent	Adsorbate	Q_max_ (mg/g)	References
**Coconut coir**	Methylene blue	15.59	[32]
**Egg shell**	Methylene blue	16.43	[33]
**Activated carbon from waste biomass**	Methylene blue	10.21	[34]
**ZnO hydrid beads**	Basic blue 41	1.0–8.0	[35]
**Polylactide/spent grain**	Malachite green	1.48	[36]
**ZnO nanoparticles**	Methylene blue	0.3428	[37]
**Coir pith carbon**	Methylene blue	5.87	[58]
**ZnO@ananas comosus waste biomass**	Celestine blue	6.52	[37]
**Ananas comosus waste biomass**	Celestine blue	5.42	[37]
**Brewery spent grain**	Malachite green	2.55	[36]
**Poultry feathers**	Malachite green	3.55	[36]
**Ananas comosus waste biomass**	Celestine blue	5.42	[37]
**MOF-5**	Merbromin	22.22	This study
**MOF-5**	Safranin O	8.19	This study

## Data Availability

The data that support the findings of this study are available from the corresponding author upon reasonable request.

## Supplementary Materials

The following supporting information can be downloaded at https://www.mdpi.com/article/10.3390/molecules29040886/s1, Figure S1: (a). EDS spectra of as synthesized MOF-5 and (b). elemental mapping of MOF-5; Figure S2: (a) pseudo second order plot of merbromin removal, (b) pseudo second order plot of safranin O removal, (c) intra-particle diffusion of merbromin removal, (d) intra particle diffusion of safranin O removal; Table S1: Statistical data for different Adsorption Kinetics; Figure S3: Different Adsorption Isotherms for Merbromin adsorption onto MOF-5 (a) Langmuir isotherm, (b) Temkin isotherm, (c) Elovich isotherm, (d) Dubinin-Radushkevich isotherm; Figure S4: Different Adsorption Isotherms for Safranin O adsorption onto MOF-5 (a) Langmuir isotherm, (b) Temkin isotherm, (c) Elovich isotherm, (d) Dubinin-Radushkevich isotherm; Table S2: Statistical data for different adsorption isotherm; Table S3: Thermodynamic parameters for dye adsorption onto MOF-5; Table S4: Various parameters in Kochbihar lake water.

## References

[1] Maheshwari K., Solanki Y.S., Ridoy M.S.H., Agarwal M., Dohare R., Gupta R. (2020). Ultrasonic Treatment of Textile Dye Effluent Utilizing Microwave-assisted Activated Carbon. Environ. Prog. Sustain. Energy.

[2] Arif A. (2020). Water Pollution and Industries. Pure Appl. Biol..

[3] Banat I.M., Nigam P., Singh D., Marchant R. (1996). Microbial Decolorization of Textile-Dyecontaining Effluents: A Review. Bioresour. Technol..

[4] Liu Q. (2020). Pollution and Treatment of Dye Waste-Water. IOP Conf. Ser. Earth Environ. Sci..

[5] Salleh M.A.M., Mahmoud D.K., Karim W.A.W.A., Idris A. (2011). Cationic and Anionic Dye Adsorption by Agricultural Solid Wastes: A Comprehensive Review. Desalination.

[6] Winckler J. (1974). Vitalfärbung von Lysosomen und anderen Zellorganellen der Ratte mit Neutralrot Vital Staining of Lysosomes and Other Cell Organelles of the Rat with Neutral Red. Prog. Histochem. Cytochem..

[7] Carvalho Pinheiro N.S., Perez-Lopez O.W., Gutterres M. (2022). Solid Leather Wastes as Adsorbents for Cationic and Anionic Dye Removal. Environ. Technol..

[8] Samal K., Mahapatra S., Hibzur Ali M. (2022). Pharmaceutical Wastewater as Emerging Contaminants (EC): Treatment Technologies, Impact on Environment and Human Health. Energy Nexus.

[9] Wall A., Board T., Banaszkiewicz P.A., Kader D.F. (2014). Chemical Basis for the Histological Use of Safranin O in the Study of Articular Cartilage. Classic Papers in Orthopaedics.

[10] Sharifpour E., Ghaedi M., Nasiri Azad F., Dashtian K., Hadadi H., Purkait M.K. (2018). Zinc Oxide Nanorod-loaded Activated Carbon for Ultrasound-assisted Adsorption of Safranin O: Central Composite Design and Genetic Algorithm Optimization. Appl. Organomet. Chem..

[11] Aronson J.K. (2016). Mercury and Mercurial Salts. Meyler’s Side Effects of Drugs.

[12] Hill S.L., Thomas S.H.L. (2011). Clinical Toxicology of Newer Recreational Drugs. Clin. Toxicol..

[13] Li M., Wang X., Porter C.J., Cheng W., Zhang X., Wang L., Elimelech M. (2019). Concentration and Recovery of Dyes from Textile Wastewater Using a Self-Standing, Support-Free Forward Osmosis Membrane. Environ. Sci. Technol..

[14] Kumari H., Sonia, Suman, Ranga R., Chahal S., Devi S., Sharma S., Kumar S., Kumar P., Kumar S. (2023). A Review on Photocatalysis Used For Wastewater Treatment: Dye Degradation. Water Air Soil Pollut..

[15] Guo J., Jiang D., Wu Y., Zhou P., Lan Y. (2011). Degradation of Methyl Orange by Zn(0) Assisted with Silica Gel. J. Hazard. Mater..

[16] Wei K., Cui T., Huang F., Zhang Y., Han W. (2020). Membrane Separation Coupled with Electrochemical Advanced Oxidation Processes for Organic Wastewater Treatment: A Short Review. Membranes.

[17] Palanisamy S., Nachimuthu P., Awasthi M.K., Ravindran B., Chang S.W., Palanichamy M., Nguyen D.D. (2020). Application of Electrochemical Treatment for the Removal of Triazine Dye Using Aluminium Electrodes. J. Water Supply Res. Technol. Aqua.

[18] Bhatia D., Sharma N.R., Singh J., Kanwar R.S. (2017). Biological Methods for Textile Dye Removal from Wastewater: A Review. Crit. Rev. Environ. Sci. Technol..

[19] Yagub M.T., Sen T.K., Afroze S., Ang H.M. (2014). Dye and Its Removal from Aqueous Solution by Adsorption: A Review. Adv. Colloid Interface Sci..

[20] Das C., Ghosh N.N., Pulhani V., Biswas G., Singhal P. (2023). Bio-Functionalized Magnetic Nanoparticles for Cost-Effective Adsorption of U(VI): Experimental and Theoretical Investigation. RSC Adv..

[21] Xu P., Zeng G.M., Huang D.L., Feng C.L., Hu S., Zhao M.H., Lai C., Wei Z., Huang C., Xie G.X. (2012). Use of Iron Oxide Nanomaterials in Wastewater Treatment: A Review. Sci. Total Environ..

[22] Das C., Sen S., Singh T., Ghosh T., Paul S.S., Kim T.W., Jeon S., Maiti D.K., Im J., Biswas G. (2020). Green Synthesis, Characterization and Application of Natural Product Coated Magnetite Nanoparticles for Wastewater Treatment. Nanomaterials.

[23] Li Y., Li L., Yu J. (2017). Applications of Zeolites in Sustainable Chemistry. Chem.

[24] Rani U.A., Ng L.Y., Ng C.Y., Mahmoudi E. (2020). A Review of Carbon Quantum Dots and Their Applications in Wastewater Treatment. Adv. Colloid Interface Sci..

[25] Liu X., Shan Y., Zhang S., Kong Q., Pang H. (2023). Application of Metal Organic Framework in Wastewater Treatment. Green Energy Environ..

[26] Aslam A.A., Irshad A., Nazir M.S., Atif M. (2023). A Review on Covalent Organic Frameworks as Adsorbents for Organic Pollutants. J. Clean. Prod..

[27] Stock N., Biswas S. (2012). Synthesis of Metal-Organic Frameworks (MOFs): Routes to Various MOF Topologies, Morphologies, and Composites. Chem. Rev..

[28] Tan Y.-X., He Y.-P., Wang M., Zhang J. (2014). A Water-Stable Zeolite-like Metal–Organic Framework for Selective Separation of Organic Dyes. RSC Adv..

[29] Chen Q., He Q., Lv M., Xu Y., Yang H., Liu X., Wei F. (2015). Selective Adsorption of Cationic Dyes by UiO-66-NH2. Appl. Surf. Sci..

[30] Wang C., Liu X., Keser Demir N., Chen J.P., Li K. (2016). Applications of Water Stable Metal–Organic Frameworks. Chem. Soc. Rev..

[31] Khan N.A., Hasan Z., Jhung S.H. (2013). Adsorptive Removal of Hazardous Materials Using Metal-Organic Frameworks (MOFs): A Review. J. Hazard. Mater..

[32] Sharma Y.C., Upadhyay S.N. (2009). Removal of a Cationic Dye from Wastewaters by Adsorption on Activated Carbon Developed from Coconut Coir. Energy Fuels.

[33] Tsai W.T., Yang J.M., Lai C.W., Cheng Y.H., Lin C.C., Yeh C.W. (2006). Characterization and Adsorption Properties of Eggshells and Eggshell Membrane. Bioresour. Technol..

[34] Karagoz S., Tay T., Ucar S., Erdem M. (2008). Activated Carbons from Waste Biomass by Sulfuric Acid Activation and Their Use on Methylene Blue Adsorption. Bioresour. Technol..

[35] Shokry Hassan H., Elkady M.F., El-Shazly A.H., Bamufleh H.S. (2014). Formulation of Synthesized Zinc Oxide Nanopowder into Hybrid Beads for Dye Separation. J. Nanomater..

[36] Muinde V.M., Onyari J.M., Wamalwa B., Wabomba J.N. (2020). Adsorption of Malachite Green Dye from Aqueous Solutions Using Mesoporous Chitosan–Zinc Oxide Composite Material. Environ. Chem. Ecotoxicol..

[37] Akpomie K.G., Conradie J. (2020). Synthesis, Characterization, and Regeneration of an Inorganic–Organic Nanocomposite (ZnO@biomass) and Its Application in the Capture of Cationic Dye. Sci. Rep..

[38] Gangu K.K., Maddila S., Jonnalagadda S.B. (2022). The Pioneering Role of Metal–Organic Framework-5 in Ever-Growing Contemporary Applications—A Review. RSC Adv..

[39] Greer H.F., Liu Y., Greenaway A., Wright P.A., Zhou W. (2016). Synthesis and Formation Mechanism of Textured MOF-5. Cryst. Growth Des..

[40] Biserčić M.S., Marjanović B., Vasiljević B.N., Mentus S., Zasońska B.A., Ćirić-Marjanović G. (2019). The Quest for Optimal Water Quantity in the Synthesis of Metal-Organic Framework MOF-5. Microporous Mesoporous Mater..

[41] Barthwal S., Jeon Y., Lim S.-H. (2022). Superhydrophobic Sponge Decorated with Hydrophobic MOF-5 Nanocoating for Efficient Oil-Water Separation and Antibacterial Applications. Sustain. Mater. Technol..

[42] Wang S., Xie X., Xia W., Cui J., Zhang S., Du X. (2020). Study on the Structure Activity Relationship of the Crystal MOF-5 Synthesis, Thermal Stability and N2 Adsorption Property. High Temp. Mater. Process..

[43] Wang S., Ye B., An C., Wang J., Li Q., Guo H., Zhang J. (2019). Exploring the Coordination Effect of GO@MOF-5 as Catalyst on Thermal Decomposition of Ammonium Perchlorate. Nanoscale Res. Lett..

[44] Iswarya N., Kumar M.G., Rajan K.S., Rayappan J.B.B. (2012). Metal Organic Framework (MOF-5) For Sensing of Volatile Organic Compounds. J. Appl. Sci..

[45] Mirsoleimani-azizi S.M., Setoodeh P., Zeinali S., Rahimpour M.R. (2018). Tetracycline Antibiotic Removal from Aqueous Solutions by MOF-5: Adsorption Isotherm, Kinetic and Thermodynamic Studies. J. Environ. Chem. Eng..

[46] Son W.-J., Kim J., Kim J., Ahn W.-S. (2008). Sonochemical Synthesis of MOF-5. Chem. Commun..

[47] Choi J.-S., Son W.-J., Kim J., Ahn W.-S. (2008). Metal–Organic Framework MOF-5 Prepared by Microwave Heating: Factors to Be Considered. Microporous Mesoporous Mater..

[48] Zhao Z., Li Z., Lin Y.S. (2009). Adsorption and Diffusion of Carbon Dioxide on Metal−Organic Framework (MOF-5). Ind. Eng. Chem. Res..

[49] Elsherbiny A.S., Rady A., Abdelhameed R.M., Gemeay A.H. (2023). Efficiency and Selectivity of Cost-Effective Zn-MOF for Dye Removal, Kinetic and Thermodynamic Approach. Environ. Sci. Pollut. Res..

[50] Wu Y., Pang H., Yao W., Wang X., Yu S., Yu Z., Wang X. (2018). Synthesis of Rod-like Metal-Organic Framework (MOF-5) Nanomaterial for Efficient Removal of U(VI): Batch Experiments and Spectroscopy Study. Sci. Bull..

[51] Tomić N.M., Dohčević-Mitrović Z.D., Paunović N.M., Mijin D.Ž., Radić N.D., Grbić B.V., Aškrabić S.M., Babić B.M., Bajuk-Bogdanović D.V. (2014). Nanocrystalline CeO _2−δ_ as Effective Adsorbent of Azo Dyes. Langmuir.

[52] Ghoohestani E., Samari F., Homaei A., Yosuefinejad S. (2024). A Facile Strategy for Preparation of Fe_3_O_4_ Magnetic Nanoparticles Using Cordia Myxa Leaf Extract and Investigating Its Adsorption Activity in Dye Removal. Sci. Rep..

[53] Di J., Ruan Z., Zhang S., Dong Y., Fu S., Li H., Jiang G. (2022). Adsorption Behaviors and Mechanisms of Cu2+, Zn2+ and Pb2+ by Magnetically Modified Lignite. Sci. Rep..

[54] Albadarin A.B., Mangwandi C., Al-Muhtaseb A.H., Walker G.M., Allen S.J., Ahmad M.N.M. (2012). Kinetic and Thermodynamics of Chromium Ions Adsorption onto Low-Cost Dolomite Adsorbent. Chem. Eng. J..

[55] Dahlan I., Wan Mazlan W.H., Mulkan A., Zwain H.M., Hassan S.R., Aziz H.A., Hasan H.Y.A., Zekker I. (2022). Modeling of Batch Organic Dye Adsorption Using Modified Metal-Organic Framework-5. Chem. Eng. Technol..

[56] Mohammadi A.A., Moghanlo S., Kazemi M.S., Nazari S., Ghadiri S.K., Saleh H.N., Sillanpää M. (2022). Comparative Removal of Hazardous Cationic Dyes by MOF-5 and Modified Graphene Oxide. Sci. Rep..

[57] Iprahim M., Farag R., Tantawe H. (2022). Removal of Dyes from Aqueous Solution Using Zinc Terephthalic Acid Metal-organic Frameworks (Zn-TPA-MOF) Based Adsorbent. Egypt. J. Chem..

[58] Kavitha D., Namasivayam C. (2007). Experimental and Kinetic Studies on Methylene Blue Adsorption by Coir Pith Carbon. Bioresour. Technol..

[59] Al-Rashed S.M., Al-Gaid A.A. (2012). Kinetic and Thermodynamic Studies on the Adsorption Behavior of Rhodamine B Dye on Duolite C-20 Resin. J. Saudi Chem. Soc..

[60] Liu X., Gong W., Luo J., Zou C., Yang Y., Yang S. (2016). Selective Adsorption of Cationic Dyes from Aqueous Solution by Polyoxometalate-Based Metal–Organic Framework Composite. Appl. Surf. Sci..

[61] Jiao C., Wang Y., Li M., Wu Q., Wang C., Wang Z. (2016). Synthesis of Magnetic Nanoporous Carbon from Metal-Organic Framework for the Fast Removal of Organic Dye from Aqueous Solution. J. Magn. Magn. Mater..

[62] Budavari S. (1989). The Merck Index: An Encyclopedia of Chemicals, Drugs, and Biologicals.

[63] Tong M., Liu D., Yang Q., Devautour-Vinot S., Maurin G., Zhong C. (2013). Influence of Framework Metal Ions on the Dye Capture Behavior of MIL-100 (Fe, Cr) MOF Type Solids. J. Mater. Chem. A.

[64] Abdi J., Vossoughi M., Mahmoodi N.M., Alemzadeh I. (2017). Synthesis of Metal-Organic Framework Hybrid Nanocomposites Based on GO and CNT with High Adsorption Capacity for Dye Removal. Chem. Eng. J..

[65] Zhao X., Liu S., Tang Z., Niu H., Cai Y., Meng W., Wu F., Giesy J.P. (2015). Synthesis of Magnetic Metal-Organic Framework (MOF) for Efficient Removal of Organic Dyes from Water. Sci. Rep..

[66] Huang L., He M., Chen B., Hu B. (2018). Magnetic Zr-MOFs Nanocomposites for Rapid Removal of Heavy Metal Ions and Dyes from Water. Chemosphere.

[67] Wang K., Li C., Liang Y., Han T., Huang H., Yang Q., Liu D., Zhong C. (2016). Rational Construction of Defects in a Metal–Organic Framework for Highly Efficient Adsorption and Separation of Dyes. Chem. Eng. J..

[68] Yao S., Xu T., Zhao N., Zhang L., Huo Q., Liu Y. (2017). An Anionic Metal–Organic Framework with Ternary Building Units for Rapid and Selective Adsorption of Dyes. Dalton Trans..

[69] Ahmad N., Younus H.A., Chughtai A.H., Van Hecke K., Khattak Z.A.K., Gaoke Z., Danish M., Verpoort F. (2018). Synthesis of 2D MOF Having Potential for Efficient Dye Adsorption and Catalytic Applications. Catal. Sci. Technol..

[70] Huo S.-H., Yan X.-P. (2012). Metal–Organic Framework MIL-100(Fe) for the Adsorption of Malachite Green from Aqueous Solution. J. Mater. Chem..

[71] Chand S., Elahi S.M., Pal A., Das M.C. (2017). A New Set of Cd(II)-Coordination Polymers with Mixed Ligands of Dicarboxylate and Pyridyl Substituted Diaminotriazine: Selective Sorption towards CO_2_ and Cationic Dyes. Dalton Trans..

[72] Zhao S., Chen D., Wei F., Chen N., Liang Z., Luo Y. (2017). Removal of Congo Red Dye from Aqueous Solution with Nickel-Based Metal-Organic Framework/Graphene Oxide Composites Prepared by Ultrasonic Wave-Assisted Ball Milling. Ultrason. Sonochem..

[73] Hazrati M., Safari M. (2020). Cadmium-based Metal–Organic Framework for Removal of Dye from Aqueous Solution. Environ. Prog. Sustain. Energy.

[74] Yang S.J., Choi J.Y., Chae H.K., Cho J.H., Nahm K.S., Park C.R. (2009). Preparation and Enhanced Hydrostability and Hydrogen Storage Capacity of CNT@MOF-5 Hybrid Composite. Chem. Mater..

[75] Yousef N.S., Farouq R., Hazzaa R. (2016). Adsorption Kinetics and Isotherms for the Removal of Nickel Ions from Aqueous Solutions by an Ion-Exchange Resin: Application of Two and Three Parameter Isotherm Models. Desalination Water Treat..

[76] Lin K., Pan J., Chen Y., Cheng R., Xu X. (2009). Study the Adsorption of Phenol from Aqueous Solution on Hydroxyapatite Nanopowders. J. Hazard. Mater..

[77] El-Nemr M.A., Yılmaz M., Ragab S., Hassaan M.A., El Nemr A. (2023). Isotherm and Kinetic Studies of Acid Yellow 11 Dye Adsorption from Wastewater Using Pisum Sativum Peels Microporous Activated Carbon. Sci. Rep..

[78] Liu Y., Liu Y.-J. (2008). Biosorption Isotherms, Kinetics and Thermodynamics. Sep. Purif. Technol..

[79] Lima E.C., Hosseini-Bandegharaei A., Anastopoulos I. (2019). Response to “Some Remarks on a Critical Review of the Estimation of the Thermodynamic Parameters on Adsorption Equilibria. Wrong Use of Equilibrium Constant in the van’t Hoff Equation for Calculation of Thermodynamic Parameters of Adsorption. *J. Mol. Liq.*
**2019**, 273, 425–434”. J. Mol. Liq..

[80] Liu Y. (2009). Is the Free Energy Change of Adsorption Correctly Calculated?. J. Chem. Eng. Data.

[81] Liu Y., Xu H. (2007). Equilibrium, Thermodynamics and Mechanisms of Ni^2+^ Biosorption by Aerobic Granules. Biochem. Eng. J..

[82] Garcia-Delgado R.A., Cotoruelo-Minguez L.M., Rodriguez J.J. (1992). Equilibrium Study of Single-Solute Adsorption of Anionic Surfactants with Polymeric XAD Resins. Sep. Sci. Technol..

[83] Das C., Singh S., Bhakta S., Mishra P., Biswas G. (2022). Bio-Modified Magnetic Nanoparticles with Terminalia Arjuna Bark Extract for the Removal of Methylene Blue and Lead (II) from Simulated Wastewater. Chemosphere.

