# Supplementary material for: Adsorptive Elimination of a Cationic Dye and a Hg (II)-Containing Antiseptic from Simulated Wastewater Using a Metal Organic Framework
Source: Molecules. 2024 Feb 17;29(4):886. doi: 10.3390/molecules29040886 (PMC10892504; doi:10.3390/molecules29040886)
Supplement: Supplementary file 1 [file molecules-29-00886-s001.zip › molecules-2864214-supplementary.pdf]

Supporting information for

Adsorptive Elimination of a Cationic Dye and a Hg (II)-Containing Antiseptic from Simulated Wastewater Using a Metal Organic Framework

Nilanjan Roy[1], Chanchal Das[1], Mohuya Paul[2], Jungkyun Im[2,3*], and Goutam Biswas[1*]

[1]Department of Chemistry, Cooch Behar Panchanan Barma University, Cooch Behar, West Bengal, India 736101

[2]Department of Electronic Materials, Devices and Equipment Engineering,

[3]Department of Chemical Engineering, Soonchunhyang University, Asan 31538, Republic of Korea

[*]**Corresponding Authors:** Dr. Goutam Biswas, Department of Chemistry, Cooch Behar Panchanan Barma University, Cooch Behar-736101, West Bengal, India; Email address: goutam@cbpbu.ac.in, and Prof. Jungkyun Im, Department of Electronic Materials, Devices and Equipment Engineering, Soonchunhyang University, Asan, Republic of Korea; Email address: jkim5279@sch.ac.kr

1. EDS analysis

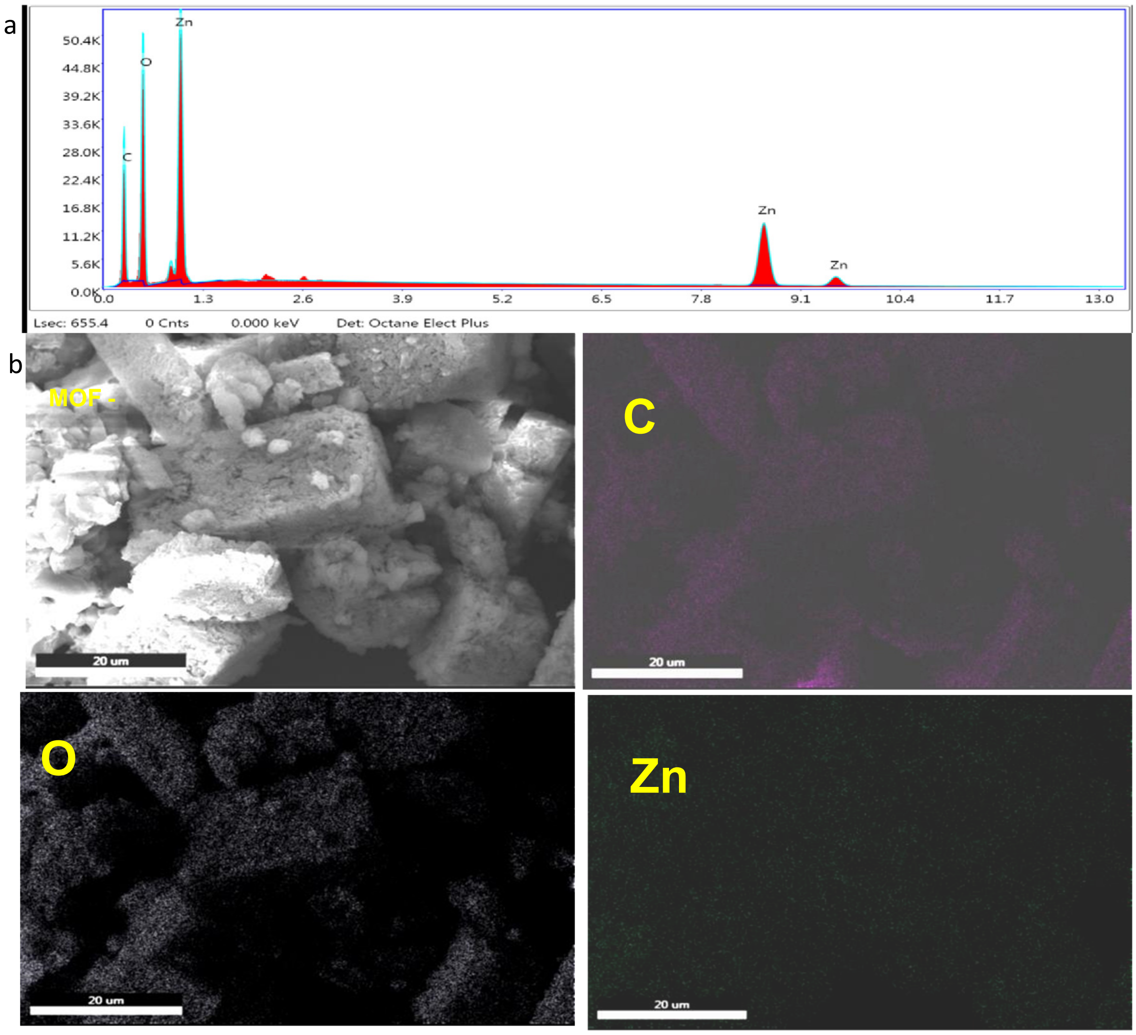

Figure S1: a. EDS spectra of as synthesized MOF-5 and b. elemental mapping of MOF-5.

2. Effect of contact time and adsorption kinetics

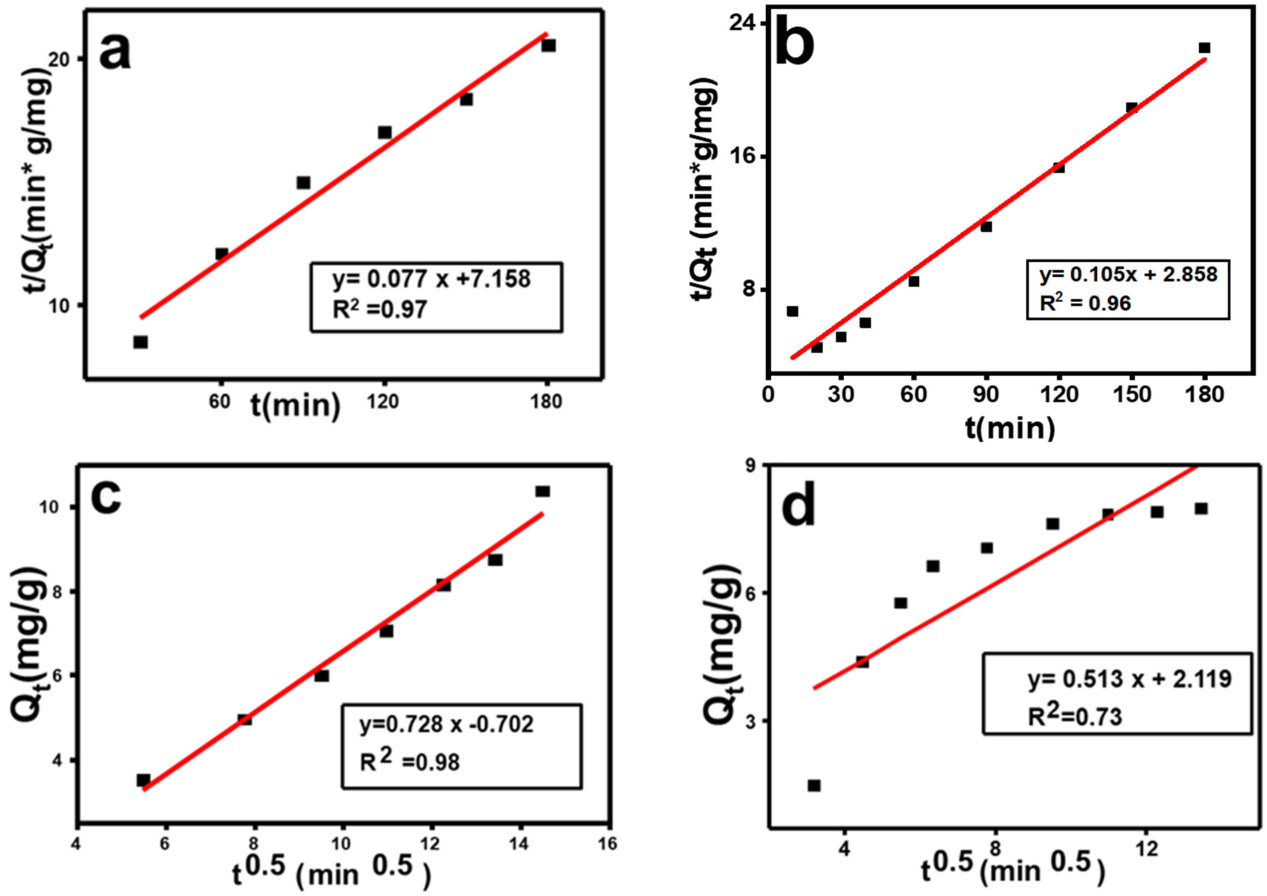

Figure S2: (a) pseudo second order plot of merbromin removal, (b) pseudo second order plot of safranin O removal, (c) intra-particle diffusion of merbromin removal, (d) intra particle diffusion of safranin O removal

Table S1: Statistical data for different Adsorption Kinetics

Adsorbate	Concentration (mg L^{-1})	order	Equation	R^2	Rate constant (k)	Qe(exp) (mg/g)	Qe(cal) (mg/g)
Safranin O	15	Pseudo 1st order kinetics	$\ln (q_e - q_t) = \ln q_e - k_1 t$	0.989	0.030	7.990	6.221
		Pseudo 2nd order kinetics	$\frac{t}{q_t} = \frac{1}{k_2 q_e^2} + \frac{t}{q_e}$	0.967	0.005	7.990	9.523
		Intraparticle diffusion	$q_t = k_i t^{0.5} + C$	0.731	0.513	7.990	--------
Merbromin	25	Pseudo 1st order kinetics	$\ln (q_e - q_t) = \ln q_e - k_1 t$	0.987	0.009	10.373	9.757
		Pseudo 2nd order kinetics	$\frac{t}{q_t} = \frac{1}{k_2 q_e^2} + \frac{t}{q_e}$	0.974	0.001	10.373	12.987
		Intraparticle diffusion	$q_t = k_i t^{0.5} + C$	0.984	0.728	10.373	----------

3. **Effect of adsorbate dosage and adsorption Isotherm**

The linear form of Langmuir adsorption isotherm is,

$$\frac{C_e}{Q_e} = \frac{1}{Q_m K_L} + \frac{C_e}{Q_m} \ldots\ldots\ldots\ldots\ldots\ldots\ldots (S1)$$

Whereas the nonlinear form is, $Q_e = \frac{Q_{max}\ K_L C_e}{(1+K_L C_e)}$ ……………………………..(S2)

On the other hand, the linear form of Freundlich isotherm is,

$$\ln Q_e = \ln K_p + \frac{1}{n} \ln C_e \ldots\ldots\ldots\ldots\ldots\ldots (S3)$$

And the nonlinear form of this isotherm is, $Q_e = K_F C_e^{1/n}$ ……………….(S4)

Where C_o = initial concentration of adsorbate in mg/L, C_e = equilibrium concentration of (adsorbate + adsorbent), Q_e = adsorption capacity in mg/g, K_L = Langmuir constant, Q_m = maximum adsorption capacity, K_p = Freundlich constant, n = separation factor. if the intensity(1/n) is within 0 to1 i.e., if separation factor >1 then only the Freundlich adsorption is favorable. [59,73–75].

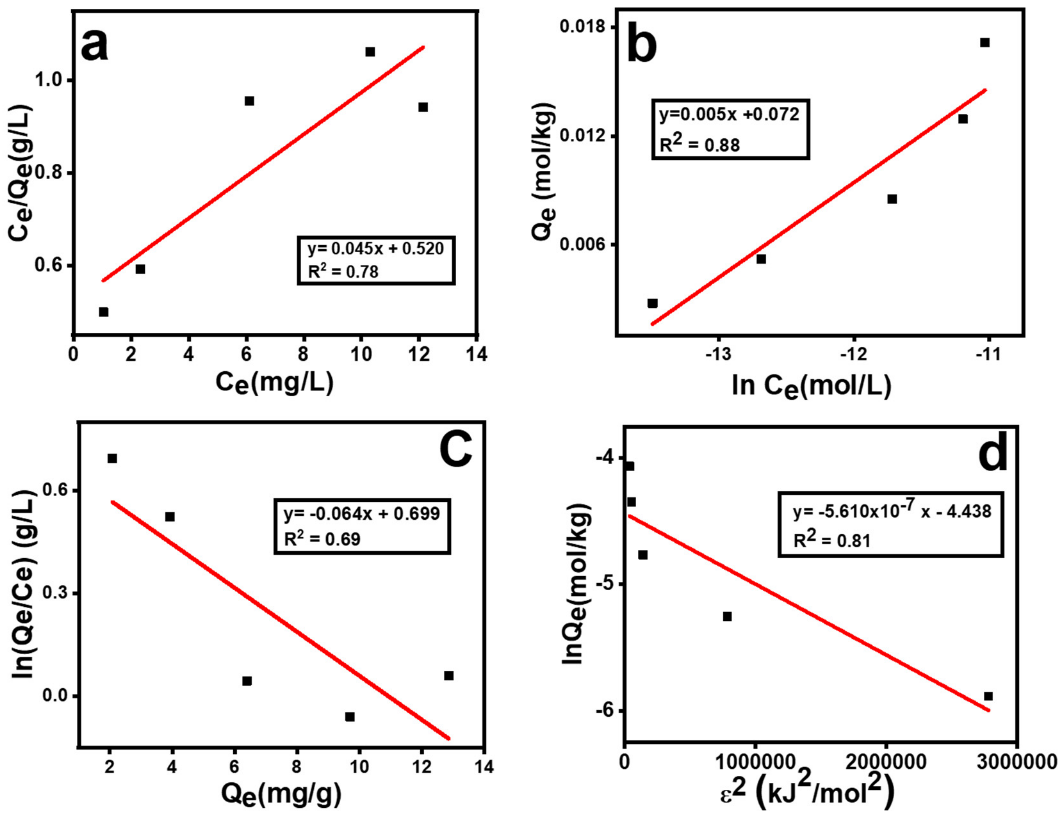

Figure S3: Different Adsorption Isotherms for Merbromin adsorption onto MOF-5 (a) Langmuir isotherm, (b) Temkin isotherm, (c) Elovich isotherm, (d) Dubinin-Radushkevich isotherm

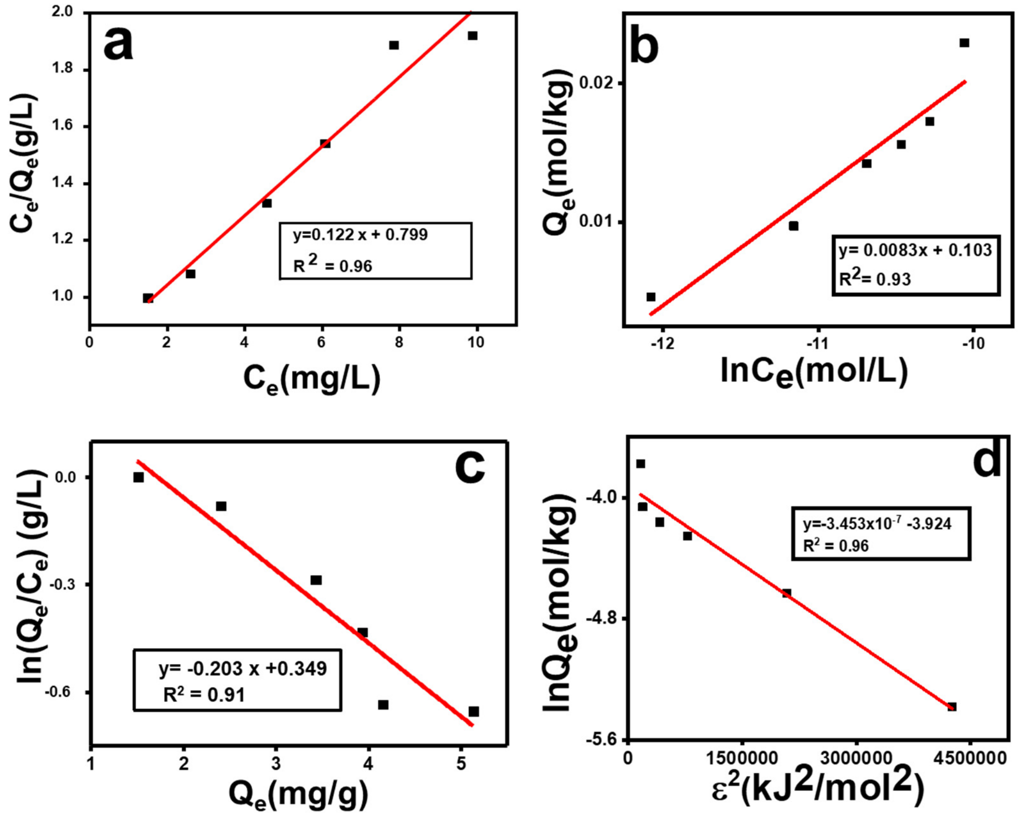

Figure S4: Different Adsorption Isotherms for Safranin O adsorption onto MOF-5 (a) Langmuir isotherm, (b) Temkin isotherm, (c) Elovich isotherm, (d) Dubinin-Radushkevich isotherm

Table S2: Statistical data for different adsorption isotherm

Isotherm	Parameter	Value	
		Merbromin	Safranin O
Freundlich ($\ln Q_e = \frac{1}{n}\ln C_e + \ln K_F$)	K_F [mg. g^{-1}(mg L^{-1}) $^{-1/n}$]	2.04	1.24
	n	1.445	1.609
	R^2_{Adj}	0.979	0.97543
	R^2	0.98425	0.98034
Langmuir ($\frac{C_e}{Q_e} = \frac{C_e}{Q_{max}} + \frac{1}{Q_{max}K_L}$)	Q_{max} (mg/g)	22.22	8.19
	R^2_{Adj}	0.713	0.957
	R^2	0.785	0.966
Temkin ($Q_e = B\ln A + B\ln C_e$)	R^2_{Adj}	0.852	0.975
	R^2	0.889	0.938
	B(kJ/mol)	0.005	0.0083
Elovich ($\frac{Q_e}{Q_m} = K_E C_e e^{-\frac{Q_e}{Q_m}}$)	R^2_{Adj}	0.596	0.898
	R^2	0.690	0.918
	Q_m	15.625	4.926
Dubinin-Radushkevich ($\ln Q_e = \ln Q_0 - \beta\varepsilon^2$)	R^2_{Adj}	0.085	0.602
	R^2	0.814	0.962
	β(mol^2/kJ^2)	5.610×10^{-7}	3.453×10^{-7}
	$\ln Q_0$	-4.438	-3.924

4. Thermodynamics of Adsorption

Table S3: Thermodynamic parameters for dye adsorption onto MOF-5.

Adsorbate	T(˚K)	ln (C_e)	ΔH (kJ/mol)	ΔS (J/mol. K)	ΔG (J/mol)
Safranin O	289	-1.92	18.77	78.35	-3866.02
	299	-1.69		76.17	-3999.79
	309	-1.44		74.14	-4133.56
	319	-1.19		72.23	-4267.34
Merbromin	289	-2.59	11.16	50.64	-3471.97
	299	-2.39		49.35	-3592.11
	309	-2.27		48.14	-3712.24
	319	-2.15		47.01	-3832.38

Table S4: Various parameters in Kochbihar lake water.

parameters	Amount	Unit
Ammonia-N	0.53	mg/l
BOD	1.80	mg/l
Conductivity	55.21	µs/cm
Dissolved O2(DO)	6.20	mg/l
Fecal Coliform	2300	MPN/100ml
Nitrate-N	0.21	mg/l
pH	7.19	Unit
Temperature (Water)	27	^{0}C
Total Coliform	8000	MPN/100ml
Boron	3.44	mg/l
Sodium	2.30	mg/l
Sulphate	6.28	mg/l
Total Alkalinity	30.00	mg/l
Total Dissolved Solids (TDS)	32.00	mg/l
Total Fixed Solids (TFS)	28.00	mg/l
Total Hardness as $CaCO_3$	24.00	mg/l
Total Suspended Solids (TSS)	10.00	mg/l

Turbidity	0.72	NTU
Calcium	8.00	mg/l
Chloride	14.67	mg/l
COD	36.72	mg/l
Fluoride	0.35	mg/l
Magnesium	0.97	mg/l
Phenolphthalein Alkanity	NIL	mg/l
Phosphate-P	0.15	mg/l
Potassium	0.80	mg/l

Reference: WBPCB (https://www.wbpcb.gov.in)
